# Update on Management of Caustic and Foreign Body Ingestion in Children

**DOI:** 10.1155/2009/969868

**Published:** 2009-11-08

**Authors:** Pietro Betalli, Alfredo Rossi, Marta Bini, Giuseppe Bacis, Osvaldo Borrelli, Cesare Cutrone, Luigi Dall'Oglio, Gian Luigi d'Angelis, Diego Falchetti, Maria Luisa Farina, PierGiorgio Gamba, Paolo Gandullia, Giuliano Lombardi, Fillippo Torroni, Claudio Romano, Paola De Angelis

**Affiliations:** ^1^Paediatric Surgery Unit, University of Padova, Via Giustiniani 3, 35128 Padova, Italy; ^2^Gastroenterology and Digestive Endoscopy Unit, NiguardaCa' Granda Hospital, Milan, Italy; ^3^Poison Control Centre, Bergamo, Italy; ^4^Pediatric Gastroenterology Division, La Sapienza University, Rome, Italy; ^5^Airways Endoscopic Surgery Unit, Padova General Hospital, Padua, Italy; ^6^Digestive Surgery and Endoscopy Unit, Ospedale Pediatrico Bambino Gesù, Rome, Italy; ^7^Pediatric Gastroenterology and Endoscopic Unit, University of Parma, Parma, Italy; ^8^Pediatric Surgery Unit, Niguarda Ca' Granda Hospital, Milan, Italy; ^9^Pediatric Gastroenterology, G Gaslini Institute, Genoa, Italy; ^10^Paediatric Gastroenterology Unit, Spirito Santo Hospital, Pescara, Italy; ^11^Paediatric Department, University of Messina, Messina, Italy

## Abstract

The following recommendations for management of caustic and foreign body ingestion in children have been developed following a multicentre study performed by the *Italian Society for Paediatric Gastroenterology, Hepatology and Nutrition (SIGENP)*. They are principally addressed to medical professionals involved in casualty. Because there is paucity of good quality clinical trials in children on this topic, many of the recommendations are currently extrapolated from adult experiences or based on experts opinions. The document represents a level 2 to 5 degree of evidence (according to the Oxford Centre for Evidence-based Medicine Levels of Evidence), gathered from clinical experience, recent studies, and expert reports discussed during a consensus conference of the Endoscopic Section of the Italian Society of Paediatric Gastroenterology Hepatology and Nutrition. This working group comprises paediatricians, endoscopists, paediatric surgeons, toxicologists, and ENT surgeons, who are all actively involved in the management of these children. Recommendations are intended to serve as an aid to clinical judgement, not to replace it and therefore do not provide answers to every clinical question; nor does adherence to them ensure a successful outcome in every case. The ultimate decision on the clinical management of an individual patient will always depend on the specific clinical circumstances of the patient, and on the clinical judgement of the health care team.

## 1. Caustic Ingestion (CI)

### 1.1. Step I: Is It Really a Caustic?

The first step of clinical approach, in case of CI, is to establish whether the substance is a real caustic or not.Strong acids have a pH of less than 2 and strong alkalis a pH of greater than 12 [1]. This information may not be enough to evaluate the real dangers of the ingested substance; chemical properties, such as concentration and hydroxy ion affinity, are parameters that can influence the severity of gastrointestinal lesions. Particular attention should be paid to caustic toxic substances such us phenols, fluoridric acid, and paraquat, since they can cause also systemic toxicity leading to potential gastric lavage. Acids and alkalis are known to produce different types of tissue damage. Acids generally cause coagulation necrosis, with eschar formation that may limit substance penetration to superficial layer of the oesophagus [2]. Alkalis in contrast combine with tissue proteins, cause colliquative necrosis and saponification, and are thought to penetrate deeper into tissues [3].

### 1.2. Step II: Is It a Confirmed or a Suspected Ingestion?

The time, modality of exposure, duration of contact, and any action taken at the scene of the incident are important in determining the management. The product container should be always collected to identify the substance. Frequently parents can only provide the brand name of the substance and if they do not bring the container to casualty, the composition may be difficult to be obtained; in this case such composition can be identified contacting the Poison Control Centre provided by most National Health Services.

The oesophageal and gastric damage depends on the volume of the ingested caustic; most children do not present any symptoms and do not have any lesion because the caustic has not really been ingested but only tasted. We would like to introduce the new concept of “accidental-deliberate” ingestion. The child drinks a consistent amount of caustic substance which is contained in a bottle of drinkable fluid (i.e., mineral water). This is most commonly the case of a rather older child who eagerly drinks a large amount of an apparently drinkable but in fact caustic substance by mistake [4].

### 1.3. Step III: Management in Casualty

Patients who present for medical care following caustic ingestion may be asymptomatic, but may also have varied signs and symptoms. Physical examination may reveal the presence of burns on the lips, chin, and hands due to manipulation of the substance.

It is discouraged to induce vomiting because this determines a further step back into the oesophagus of caustic with increased risk of injury or ab-ingestiis [5] and the administration of large volumes of liquid in order to dilute the caustic as this can lead to excessive production of gastric juices increasing the risk of vomiting. The conviction that an alkali should be neutralized with an acid and vice versa is wrong and can also cause an exothermic reaction resulting in worsening of the lesion. The use of activated charcoal is ineffective [6].

At the moment there are no certain biochemical markers of injury, although some parameters such as neutrophilic leukocytosis and metabolic acidosis can give an indication on the severity of the patient's clinical condition. Hemogasanalysis is useful for defining the severity of lesions [7]. A state of metabolic acidosis (pH < 7.22), arising mainly from tissue necrosis, is an indicator of serious damage with negative prognosis. Regarding neutrophilic leukocytosis, some authors [8] consider them among the indices of negative prognosis, in other studies [9, 10] this is not considered reliable; the significance in the timing and execution of these investigations remains to be determined.

### 1.4. Step IV: The Endoscopy

The panel's opinion, according with the literature, is that endoscopy can be performed even earlier not later than 24 hours [5, 6]. In case of high volumes of caustic substance (voluntary ingestion or “accidental-deliberate”) the investigation should be carried out as early as possible.

The results of a recent observational Italian study [4] demonstrate that the incidence of serious esophageal lesions without any early signs and symptoms is very low and that endoscopy could be avoided. Endoscopy might therefore be considered mandatory for asymptomatic patients only if presenting with a history of “accidental-deliberate” CI.

The risk of severe damage to the esophagus increases proportionally with the number of the patient's signs and symptoms. Endoscopy is warranted in all symptomatic children.

Endoscopy is also of great importance for monitoring the injuries and their possible stenotic evolution (Table 1). There are no controls for endoscopic lesions grades 0 to 1. In second degree injury, it can be justified to undertake ambulatory clinical control after 4 weeks. To deal with the most serious injuries (grade III), some authors suggest the endoscopic control after about 10 days [11, 12] while almost all authors and the panel consider a useful radiological control 30 days after the ingestion and an endoscopy 4–6 weeks after the ingestion [11–15].

The involvement of the larynx during CI is usually considered rare. The commitment of respiratory function presents different values between the adult (1%) and the child (2.5%) [16–18]. The severity of injuries seems to be related to the methods of exposure (accidental or intentional) and to the volume ingested. The laryngeal injury is never isolated but constantly associated with oesophageal injuries and are always severe (grade III) [19]. The laryngoscopic evaluation is indicated by symptoms of dysphonia and/or dyspnoea.

### 1.5. Step V: The Medical Treatment after Endoscopy

The medical treatment has a supportive role, and there is no evidence of any direct effect on injuries and the prevention of stenosis [13]. De Jong et al. [20] and Mamede and de Mello Filho [21] demonstrated the effectiveness of the use of corticosteroids (CSs) in reducing the extent of stenosis and the need of endoscopic treatment. The CSs seem to reduce the formation of granulation tissue and the proliferation of fibrotic tissue [14]. In association with CS, both in grade II and grade III lesions, the administration of proton pump inhibitors (0.7–3.5 mg/kg/day) appears to have a positive effect and prescribing a semifluid diet for at least 78 hours [14]. Dexamethasone (1 mg/kg/day) appears to be more effective than Prednisone (2 mg/kg/day) to reduce the degree of stenosis [5, 6, 15, 20]. De Jong et al. [20] instead indicate how the use of high doses CS could lead to an increased risk of perforation in the paediatric population and in patients with grade III injury. Anderson and Rouse [22] in a prospective study found no statistically significant differences in favour of patients undergoing steroid therapy compared to the risk of forming stenosis. Despite the absence of conclusive data, the maximum efficacy is achieved when treatment is started within the first 8 hours [23]; in case of grade III lesions it is suggested Dexamethasone 1 mg/kg/day for 3 days and gradually weaned at 10 days [13]; however, further prospective studies are needed for demonstrating the efficacy of the CS in changing the natural history and reducing the risk of stenosis.

The Nasogastric tube can be useful in the most serious cases to avoid the formation of adhesions between the oesophageal wall preventing the oesophageal stenosis [24]. Controversial is the use of antibiotics in the treatment of serious injuries. The only description in the recent literature is the one that requires the use of Ampicilline 50–100 mg/kg/day for 10 days [13, 25].

Medical therapy based on endoscopic grading of lesions is in Table 2 summarized.

### 1.6. Step VI: Endoscopic Management of Oesophageal Stenosis

Two attitudes regarding the treatment of oesophageal strictures are described in the literature. The first is conservative and involves a quick execution of oesophageal endoscopic dilatation. The second, less common, provides the consolidation of stenosis, possibly around an NG-tube, and the execution of surgical oesophageal replacement. According to the majority of the literature [26–31], the panel considered that in subjects with clinical and socioeconomic favourable situations, the conservative attitude gives better results in terms of quality of life, morbidity, and mortality.

**When to Start with Dilatations?**

Early (from fourth to sixth weeks after the ingestion) start of expansion in order to avoid stenosis tortuous and slim. Various authors [22, 30, 31] agree that the treatment should be started with expansion after the healing of the mucosal lesions, which usually occurs about 3 weeks after the ingestion.

**Which Kind of Dilatation?**

The panel suggests the pneumatic dilatation in the treatment of stenosis of the recent appearance and semirigid (Savary) in stenosis with scarring consolidated results.

**Timing and size of Dilatations**

On this point there is no correlation. The panel, based on its experience and the literature [22], suggests to increase the size of 2–4 mm per expansion. Regarding the timing of expansion, it is generally accepted at an interval between 3 and 6 weeks on first approach. The following timing must be based on signs and symptoms.

**Mitomycin C**

It has been proposed to use topical Mitomycin C, applied after dilatation, in order to slow the fibroblastic proliferation and relapse of stenosis. It is suggested at a concentration of 0.1 mg/mL and 5 minutes of application [32].

**Interruption of Dilatation Program (Refractory Stenosis)**

The panel believes, according to the literature [33] that a stenosis is defined refractory [34], when it is not possible to dilate up to a diameter that reduces dysphagia.

## 2. Foreign Body (FB) Ingestion

### 2.1. Step 1: Emergency Assessment 

FB ingestion is a quite common event in childhood, with the highest peak incidence between 1 and 2 years of life. FB ingestion is always an accidental event (93% of cases) except for patients with neurological defects or psychiatric diseases. An FB usually passes through the digestive tract without damage; among paediatric patients, approximately 80% eliminate the FB naturally over a week [35]; 20% require an endoscopic removal; 1% undergoes surgery for removal itself or for the presence of complications [35].

The different types of ingested FB can be schematically listed, both on the basis of morphological categories, both in relation to their potential hazard:

- Foods: meat boli, large pips, bones, cartilage, bones of fish).
- Objects: harmless (e.g., coins or similar) or dangerous (pins, sticks, paper clips, long or bulky objects)
- Toxiccontainers (disk batteries, items containing lead, containers of drugs).

Children may be asymptomatic but may also present scialorrhea, food refusal and odynophagia, hematemesis, vomiting, dysphagia, drooling, cough, wheezing, and dyspnoea. The symptoms depend on the location of the FB and its characteristics.

If the FB has exceeded the lower oesophageal sphincter, the patient is usually asymptomatic, as occurs in approximately 50% of children [36].

In case of ingested substance containing lead, symptoms of acute intoxication may occur, involving the digestive tract as well as the renal function and the nervous system [37].

The main goal of the physical examination of the patient should be to highlight signs of damage or oesophageal perforation, as the subcutaneous emphysema of the neck (crackles and/or hyperaemia of the neck region). If the patient presents with inspiratory stridor, we are certainly facing an obstruction of the upper airway. Unilateral wheezing or a localized reduction of murmur vesicular can suggest lower airway obstruction. The abdomen should always be examined to detect the presence of any evidence of peritonitis and in these situations the opinion of the surgeon is crucial [38].

The radiological examination is a very important and often decisive step in the assessment of a patient with an ingested FB. A chest X-ray without contrast is generally sufficient to verify the presence and localization of radiopaque FB.

If radiopacity of the FB is questionable, it can be very useful to assess a twin object, when present. Radiolucent objects can escape the first detection; the use of an adequate contrast can be useful. The X-ray examination should be performed, if possible, at the erected station, including the neck, the chest (anterior and lateral projection), and the abdomen [39]. The radiological examination is also essential in defining the diagnostic of possible complications, such as pneumomediastinum or pneumoperitonaeum [40].

### 2.2. Step 2: Indications for Emergency and Endoscopic Timing

The indication and timing of endoscopic removal in the management of paediatric patients who accidentally swallowed an FB depend on many factors: (1) the type of FB; (2) the site in which the FB is trapped in; (3) the general conditions of the patient and the clinical picture; (4) the availability of logistic supports and adequate equipment for the endoscopic procedures.

Indications and timing of FB endoscopic removal are in Table 3 summarized.

### 2.3. Coins

Coins represent the most frequently ingested FB among children; in 2003, 92 166 cases in US were documented [41, 42].

As stated before, risk factors conditioning the FB impact are, stenosis or malformations, previous surgical oesophageal interventions or contemporary ingestion of more than one coin. In case of proximal trapping patients can present with serious symptoms (wheezing, dysphagia, dyspnoea). Children can be unable to control their secretions and present higher risk of aspiration (ab-ingestis). Coins localized in the middle and lower oesophagus give usually less important symptoms (light pain, possible vomiting, rarely hematemesis). Endoscopic removal is to be considered mandatory and urgent for all coins trapped at the upper oesophagus but also for symptomatic coins anywhere localized in oesophagus.

There is no agreement in the literature concerning the management of asymptomatic patients [43, 44]. In 2005 a prospective randomised trial [43] which considered only asymptomatic and risk-free patients with esophageal coin localization demonstrated good probability (about 30%) of spontaneous passage towards gastric cavity. Therefore, in case of healthy asymptomatic children presented with esophageal localized coins, the panel suggests clinical observation for some hours, until 16, according to the only RCT [43], and repeated X-ray and endoscopic removal if spontaneous transit fails. Asymptomatic patients, with coins in the gastric cavity, can be discharged and controlled by RX examination one month later. Endoscopic removal will be considered for patients with coins still in the stomach. Endoscopic removal is also indicated for patients even asymptomatic but when they present with other pathological conditions, such as Crohn's disease or past surgical interventions which could limit FB progression through the digestive tract.

### 2.4. Batteries

Stylus-shaped batteries (SSBs) generally do not release toxic compounds and therefore should be considered as simple FB. Their endoscopic removal will depend on their size and localization. Nevertheless, particular warning must be given to the quality certification of the batteries themselves. Disk batteries (DBs) commonly used for watches or cameras are of different types, according to their different electrolyte system (Carbon-Zinc, Alkaline, Silver oxide, Lithium, etc.). In any case the leakage of their contents can produce mucosal damage or systemic toxicity.

The dangerous power of DB is determined by two main factors: local activation of electric current and possible DB splitting, followed by release of caustic compounds with consequent local or systemic toxicity.

DB splitting, apart from traumatic causes such as a bite, is related to the corrosive power of the acid gastric juice which may destroy the plastic seal connecting the two poles of the battery, with subsequent leakage of toxic compounds and caustic substances. Therefore the “critical time” is represented by the DB staying in the gastric cavity; while as the battery passes the pylorus and it is still remaining intact the risk of splitting decreases. Nevertheless the risk of systemic toxicity, concerning above all DB containing mercury, is theoretical. In fact no deaths were registered by the National Button Battery Ingestion Hotline among 2320 cases collected in 7 years [45, 46].

The following recommendations are suggested by the panel.

- DB in the oesophagus: endoscopic retrieval as soon as possible.
- DB in the stomach: endoscopic retrieval if symptoms are present, if X-ray shows lack of integrity or splitting, if the battery has failed to pass the pylorus at 48 hours.
- DB in the duodenum: no indication for retrieval: 85% of DB disappear spontaneously within 24 hours [47].

Washing bowel solutions can be used to facilitate the DB's progression through the bowel itself [48].

### 2.5. Magnets

Singly ingested magnets do not cause specific problems and have to be considered as not dangerous foreign bodies. Two or more magnetic objects lying into different intestinal loops may produce a considerable attraction force with consequent crushing of intestinal walls. Severe damages such as ulcerations, perforation, haemorrhage, and fistulas can occur [49, 50]. According to the literature, the panel suggests the following:

- urgent retrieval for multiple magnets when localized into the stomach,
- close clinical followup if they have passed the pylorus,
- immediate surgical approach if patient becomes symptomatic [51].

### 2.6. Leaded Objects

Given the wide diffusion of improperly controlled objects, the possibility of ingestion of FB containing toxic amount of lead has been reported [44, 48, 52].

In 1991 the Centre for Disease Control and Prevention stated that lead blood level greater than 10 mcg/dl must be considered toxic for paediatric age [48].

Clinical syndromes usually appear when lead level raise over 45 mcg/dl: abdominal pain, haemolytic anaemia, renal and hepatic failure, acute encephalopathy, chronic and irreversible neurotoxicity.

In case of ingestion of lead containing objects the following management is suggested:

- X-ray evaluation of the localization,
- urgent endoscopic retrieval, even in asymptomatic patient,
- blood test for lead level, if > 45 mcg/dl start with 2-3 mesodimercaptosuccinic acid Succimer 10 mg/Kg 3 times a day for 5 days, than 10 mg/Kg bid for 2 weeks (protocol approved by FDA for paediatric population),
- PPI's administration in order to decrease the lead leakage from the object if it is still gastric-located.

If endoscopic retrieval cannot be performed, the FB must be closely monitored by X-ray and serial tests for blood lead concentration must be done. We finally underline that in case of ingestion of an unknown metallic FB, lead blood levels must be checked.

### 2.7. Step 3: Endoscopic Techniques

General anaesthesia with airway intubation is the best approach for the removal of foreign bodies since it guarantees the following:

- airway protection,
- complete muscular relaxation,
- Total unconsciousness in order to prevent patient's reactivity,
- easy switch into open surgery when endoscopy is unsuccessful or in case of complications.

Almost 20% of foreign body ingestion require endoscopic removal [53].

The procedure is performed using a flexible video endoscope which should have the highest possible diameter in order to be able to use all the available devices. If an object identical to the ingested one is available, it is very useful to test on it the grip strength as well as the easy release mechanism of different devices (Forceps, Dormia basket, Net baskets, Polipectomy snares). Never use a device that could stick to the ingested object since in case the removal becomes too difficult or risky, it might be impossible to extract the endoscope.

Patient should possibly lay on the left side with the head not too flexed on the neck in order to reduce the sharpness of the angle of the upper oesophageal sphincter level and facilitate the foreign body retrieval.

Changing patient's position should always be possible in case it gives a better endoscopic access to the ingested object.

Endoscopic intubation must be done under visual control especially if the foreign body is located in the oesophagus to prevent lesions of the oesophageal wall.

If it is not stuck, and there is no previously known oesophageal pathology, the foreign body can be gently pushed along the oesophagus into the gastric lumen where all the endoscopic manoeuvres are easier and safer. Mild insufflation is useful to detach the object from the oesophageal wall. An adequate mucosal protection device is recommended in case of wounding objects.

In case of voluminous bodies a disinflation of the tracheal tube balloon could make the retrieval easier.

In case of elongated foreign bodies the major diameter of the object should be parallel to the endoscope's positioning the wounding extremity.

Cardias can be a difficult point to pull the object through. The advice is to not immediately force the pulling but to maintain a gentle traction and wait until the sphincter pressure diminishes and allows the object to be pulled backwards smoothly.

Food boli impaction in the paediatric age is usually due to a pre-existent oesophageal pathology (i.e., previous caustic ingestion, surgery for oesophageal atresia) [53, 54]. When the bolus is strongly stuck, it should not be blindly pushed down but it should be removed in one shot or in a piecemeal way. For this purpose, a very useful device is the small plastic cylinder designed for variceal rubber bands. When the endoscope's tip, with the device on it, leans to the bolus while maintaining continuous aspiration, the food gets “sucked” into the plastic cap and can be easily retrieved.

Multiple magnet ingestion represents a serious emergency. Sticking together in the bowel lumen, magnets can cause an obstruction. Even if they are in different bowel loops they can still attract and stick together causing ischaemia and possible perforation of the pinched enteric wall area [55–57].

### 2.8. Step 4: The Staff

An urgent endoscopy, such as a foreign body retrieval, cannot be set up without a very trained organization. Most urgent paediatric endoscopies are performed in the operative surgical room, whose quality levels and operative standards are usually well defined.

The staff must be trained by an expert endoscopist, at least one endoscopy-dedicated nurse who has adequate training in the management of instruments and accessories, the anaesthetists with a nurse for anaesthesia assistance.

### 2.9. Step 5: The Surgeon's Role

There are no clinical trials or reports with strong recommendations on this topic, thus the conditions for the surgeon's involvement in the present paper are stated on the basis of panel opinion.

Emergency surgical procedure must be considered if complications occur during endoscopic removal [58].

- Delayed surgical treatment should be considered in cases of FB being too bulky or potentially harmful to predict a complete and safe endoscopic removal. This is the case, for example, of bezoars, whose evacuation requires gastrostomy even in cases not complicated by pica [59, 60], or sharp/cutting objects, with at least one dimension unsuited to the oesophageal diameter [61].
- Patients presenting with multiple magnet ingestion should be timely managed by surgical approach if the magnets have already passed the duodenum [56, 62–65].
- Laparotomy must be also considered in cases in which FB progression through the gut has failed and X-ray examination shows that it is blocked [52].
- Finally, the literature emphasizes the need for surgical evaluation in children whose malformative pathologies are known [66] as well as surgical sequela that could determine transit difficulties, and in any case, when the patient comes late to the observation, for unnoticed ingestion or unclear history [67].

All indications for surgery after FB ingestion are in Table 4 summarised.

## Figures and Tables

**Table 1:** Endoscopic classification of oesophageal burns.

Endoscopic findings	Extension of lesions
No lesions	
Erythema	
Pseudomembrane	Not circumferential
Ulceration/necrosis	Not circumferential
Pseudomembrane	Circumferential
Ulceration/necrosis	Circumferential

**Table 2:** Medical therapy in caustic ingestion.

	Grade I	Grade II	Grade III
Corticosteroids	No	No	Yes
PPI	No	Yes	Yes
Antibiotics	No	No	Yes

**Table 3:** Indications and timing for foreign body endoscopic removal.

Localization	Type of FB	Timing of endoscopy
Crycopharinx/impact on stenosis	Any type	Emergency
Oesophagus	Batteries/dangerous or toxic-containing FB	Urgency
Oesophagus	Harmless FB, round-shaped—symptomatic patient	Urgency
Oesophagus	Harmless FB—asymptomatic patient	Delayed urgency, after some hours and new X-ray
Stomach	Dangerous/toxic-containing FB	Urgency
Stomach	Batteries	Delayed urgency max 48 hours
Stomach	Harmless FB in asymptomatic patient	Election (discharge and first X-ray 4 weeks later, if elimination by stools failed)
Duodenum	Dangerous FB	Urgency
Duodenum	Harmless FB	No indication
Any location	Lead containing DB	Urgency

**Table 4:** Foreign body ingestion, when the surgeon should be involved.

Emergency/urgency	Severe clinical presentations	Serious respiratory problems	dyspnoea
			cyanosis
		Acute abdomen	perforation
			occlusion
		Severe haemorrhage	
	Unexpected situation or complication occurring during endoscopic removal		

Election/delayed	FB too big or potentially dangerous	Bezoars	
		FB big, sharp, cutting	
		Multiple magnets	
	Documented failure of progression through the gut		
	Patients with congenital or acquired pathologies which could cause transit difficulties		
	Late observation for not noticed ingestion or not clear anamnesis		
